# Supplementary material for: Perspectives of teachers of students with intellectual disabilities on the role of educational policies and systems in using Universal Design for Learning: A qualitative study
Source: PLoS One. 2025 Sep 26;20(9):e0333432. doi: 10.1371/journal.pone.0333432 (PMC12468860; doi:10.1371/journal.pone.0333432)
Supplement: Attachment — Submitted filename: Response to Reviewer.docx [file pone.0333432.s002.docx]

**Editor**

| Reply | Editor Comment | No. |
| --- | --- | --- |
| The percentage has been added to the table. | "Table 3. Summary of the allocation of subthemes to main themes and the percentage of participants who discussed each subtheme" Where is percentage? | 1 |
| Themes are defined, specifically what they represent.  3.1. Attitudes toward UDL  (This theme encompasses participants’ opinions and perceptions regarding the concept of UDL and its application with their students)  3.2. Challenging educational policies and systems in the use of UDL  (This theme highlights the barriers and systemic constraints that hinder the implementation of UDL by teachers)  3.3. Solutions to address the challenges of educational policy and systems in the use of UDL  (This theme focuses on the facilitators that support teachers' effective adoption of UDL) | Attitude toward UDL  Define your themes, particularly what they represent. This will help the reader see whether the subthemes and quotations lie within this representation or not. For example, what ideas are to be cosidered as "Attitude toward UDL"? | 2 |
| This section has been revised and the themes have been changed to:  Subthemes have been changed:   - conceptualization - Positive attitude | In your thematization, Atitude may suggest teachers disposition or inclination to use or resist UDL. In here expected subthemes could be Positive attitude and interest towards using the method or resistance or appreaciation, and may be conceptualization. However, effect of using UDL principles should come under a diffeerent theme. Besides, you are not interested in evaluating the effects of the method, rather teachers perspectives. I suggest this section needs to be revisited. | 3 |
| Quotes have been added regarding how the principle can be adapted to context. | the thmes and subthemes are few and question the holistic understanding of teachers perspectives who implement UDL. for example, did they not say anything about how the principle could be adapted to your context? | 4 |
| The theme has been changed to a more relevant and appropriate one for the arrangement of findings and discussion:  The vagueness of the concept of UDL and the positive attitude towards its principles | The discussion section begins with effect of adopting UDL principles. If this need be emerge as a theme, it has to come at the end. this needs re-strcturing the "findings" section and discussion section correspondingly. | 5 |
| Yes, done. | Writing: "Challenging in the use of UDL" line 445. is it to mean Challenges? | 6 |
| A section has been added highlighting the contribution to the theory or framework. Similarly, through practical implication, it highlights how it has been successfully applied and ensured quality learning. | You examinied perspectives of teachers in adopting UDL framework. I wish there could be a theretical and practical implication of the findings. By theoretical implication, you may highlight any contribution to the theory or framework. similarly, with practical implication, you may highlight how it can be successfully implemented and ensure quality of learning. | 7 |
| done | Please adress reasonable comments of the reviewers. | 8 |

**Reviewer1**

| Reply | Reviewer Comment | No. |
| --- | --- | --- |
| A number of references published after 2020 were consulted, including:  Reference Nos. 4, 6, 9, 17, 19, 40, 48  Two new references were also added:  (Kapil et al., 2024), (Anderson, 2022). | UDL research development Please note that UDL research has significantly evolved since 2020. Earlier sources, although valid, should not be presented as representative of the current theoretical framework. | 1 |
| Done.  Added in the introduction | Inclusive cultures When discussing practices and policies, it would be appropriate to also mention inclusive cultures, referring to the three dimensions promoted by the Index for Inclusion (Booth & Ainscow, 2002). | 2 |
| Done.  Modified in the Methodological Approach section. | Use of first person To maintain an academic writing style, it is recommended to avoid first-person formulations (e.g., in the “Methodological Approach” section). | 3 |
| The inclusion criteria were teachers who: (1) had more than five years of experience teaching students with ID; (2) taught at the intermediate level within the intellectual education programs attached to general education, and (3) were willing to participate in interviews. Male teachers were excluded from the study for practical reasons  The phrase "relevant information" has been removed. | Participant selection criteria On what basis (besides gender) were participants selected? What does “relevant information” (line 180) mean in this context? The selection process, as formulated, may introduce a potential bias. | 4 |
| in this study, I have intentionally foregrounded individuals with intellectual disabilities, given their notable underrepresentation in qualitative research examining the Universal Design for Learning (UDL) framework. As Rao et al. (2017) observed, existing research on UDL implementation rarely includes students with intellectual disabilities as direct participants. Instead, it predominantly reflects the perspectives of students with other disabilities or those without disabilities, resulting in a fragmented and heterogeneous knowledge base concerning effective UDL-informed curricula and interventions tailored for students with intellectual disabilities.  My study also underscores the importance of extending individualized attention to all students within the classroom environment—not exclusively to those with intellectual disabilities. This inclusive approach is reflected throughout the manuscript, including:  - Introduction: References 4, 5, 10  - UDL: References 11, 12, 13, 17  - UDL and the promotion of inclusion for students with ID: Reference 22 | Generalization of accommodations It would be helpful to make more explicit the principle of generalising special attention to all students in the class, not just those with ID: "what is essential for some becomes useful for all". | 5 |
| This was done through the following references: 2-9-11-22-23-24-25-33-34 | Article structure Some references or sections currently found in the “Discussion” would be more appropriately placed in the “Introduction.” | 6 |

**Reviewer #2**

| Reply | Reviewer Comment | No. |
| --- | --- | --- |
| Done. | - Page 7, under methodological approach, you say "I conducted..." but then on page 8, you move to describing yourself as "the researcher" at lines 179 and 194. I would stay consistent - and I personally much prefer first person instead of "the researcher...." | 1 |
| Done. | Page 9, line 206, you say "we interviewed..." Who is the we? Previously you were saying "I" so I got confused here. | 2 |
| Done. | Table 3, page 11 - check your numbering. It looked inaccurate to me | 3 |
| Done. | Page 13, UDL Concept - can you expand on their knowledge of UDL? You really only have one paragraph, and then a lengthy quote. That doesn't really help me understand their knowledge of UDL at all. | 4 |
| Done. | - Page 15, line 317, and throughout until the end - you use the word "confirm" a lot. Qualitative research doesn't seek to "confirm" anything. I would find a different word to use throughout. | 5 |
| - Administration: Refers to internal school management personnel, including the school principal and deputy principal. This body is responsible for the execution of school operations and overseeing instructional practices within the school.  - Supervision: Refers to external educational personnel appointed by the Ministry of Education. Their primary role is to visit schools, observe classroom instruction, and evaluate teaching performance. Supervision operates independently from the school’s administrative structure and often serves as a liaison between policy implementation and classroom practice. | Page 16, what is the difference between supervision and administration? | 6 |
| Participants emphasized that the school principal functions primarily as an executive body with no autonomous authority to initiate changes or issue decisions without prior approval from senior officials at the Ministry of Education. This restricted role limits the ability to respond promptly to pedagogical innovations.  One notable example concerned teachers seeking to apply the UDL principle of “multiple means of representation and engagement” through experiential learning—such as organizing field trips that allow students to participate in practical, real-world applications of lesson content. In such cases, obtaining formal approval entails navigating prolonged bureaucratic procedures at the ministerial level, often taking several months. Given that such delays extend into the academic semester’s final stages, the opportunity for implementation may be lost entirely. Participants suggested that had the school principal possessed the authority to approve such initiatives independently, the process could be expedited significantly—potentially requiring only a few days. | Page 16, "...which affected many aspects adversely" - explain this more in depth. | 7 |
| The first quote highlights systemic barriers related to curriculum policies that hinder equitable access to the general curriculum and contribute to the persistence of the private curriculum. Specifically, it points to the rigid requirement of implementing a unified curriculum with standardized objectives for all students, irrespective of their individual backgrounds and learning profiles. This requirement directly contradicts the core philosophy of Universal Design for Learning (UDL), which emphasizes the uniqueness of each learner and advocates for personalized instructional goals.  Moreover, participants noted that previous approaches adopted by the Ministry of Education were more flexible and better aligned with UDL principles. These earlier measures granted teachers the autonomy to tailor learning objectives according to students' abilities and needs. In contrast, under current regulations, uniform objectives and lesson plans are mandated for all students. Such limited and simplified objectives fail to meet the needs of students with advanced capabilities, thereby impeding their cognitive development and depriving them of intellectually stimulating challenges.  The second quote further illustrates the limitations imposed on curriculum modification. It emphasizes that teachers are required to implement a standardized private curriculum for all students, with little or no room for adjusting objectives based on learner diversity within the classroom. Any attempt to modify the official curriculum—such as documenting alternative learning goals in the daily preparation file—is viewed as a violation of current educational regulations. This strict adherence undermines differentiated instruction and stands in contrast to the UDL framework, which inherently rejects the notion of a one-size-fits-all curriculum. | Page 17, How doe the two lengthy quotations relate to UDL? I don't see the relationship there at all. | 8 |
| It was replaced.  Participant interviews revealed that the commitment of decision-makers to the framework—demonstrated through its inclusion in official regulatory guidelines, continuous emphasis on its application, and mechanisms for accountability—constitutes a critical factor in securing teachers' engagement with it. Additionally, supervisory follow-up plays a central role in reinforcing its implementation, ensuring that educators adhere to the framework in practice. | Page 17, "The results indicated the positive effect..." In what way do the results indicate this? Be more clear | 9 |
| Participants emphasized that effective implementation of Universal Design for Learning (UDL) principles necessitates an increase in the current educational budget. This is due to the demand for diverse technical and material resources that support multiple modes of presentation, engagement, and expression. Additional requirements such as field trips, reinforcement activities, and other inclusive educational experiences further compound these needs. Several participants noted that teachers, operating without sufficient institutional support, are often unable to meet these demands alone—with some even reporting that they personally subsidize such resources from their own salaries. | Page 18, Funding programs...What is the relation in this paragraph between budget and UDL? This was unclear to me. | 10 |
| The previously cited quote has been substituted with one that explicitly connects budgeting considerations to the principles of Universal Design for Learning | Page 19, Quotation at the top of the page - how does this relate to UDL? Make a connection between budget and UDL. | 11 |
| Done | Pages 20-22, watch for use of the word "confirm" | 12 |
| Done  It has been replaced | Page 22 - This might be personal preference, but I prefer "findings" instead of "results" for qualitative research | 13 |
